# Association Between Antiarrhythmic, Electrophysiological, and Antioxidative Effects of Melatonin in Ischemia/Reperfusion

**DOI:** 10.3390/ijms20246331

**Published:** 2019-12-15

**Authors:** Ksenia A. Sedova, Olesya G. Bernikova, Julia I. Cuprova, Alexandra D. Ivanova, Galina A. Kutaeva, Michael G. Pliss, Ekaterina V. Lopatina, Marina A. Vaykshnorayte, Emiliano R. Diez, Jan E. Azarov

**Affiliations:** 1 Department of Biomedical Technology, Faculty of Biomedical Engineering, Czech Technical University in Prague, Sitna sq. 3105, 27201 Kladno, Czech Republic; sedova.ks@gmail.com; 2 Institute of Physiology, Federal Research Centre, Komi Science Centre, Ural Branch of Russian Academy of Sciences, Pervomayskaya st. 50, 167982 Syktyvkar, Russia; m.vaykshnorayte@mail.ru (M.A.V.); j.azarov@gmail.com (J.E.A.); 3 Department of Health Care Disciplines and Population Protection, Faculty of Biomedical Engineering, Czech Technical University in Prague, Sportovcu st. 2311, 27201 Kladno, Czech Republic; efremyul@fbmi.cvut.cz; 4 Department of Human and Animal Physiology, Lomonosov Moscow State University, Leninskiye gory, 1, 12, 119234 Moscow, Russia; ashka02@yandex.ru; 5 Department of Physiology, Institute of Medicine of Pitirim Sorokin Syktyvkar State University, Starovskii st., 55, 167001 Syktyvkar, Russia; g.kutaeva@gmail.com; 6 Institute of Experimental Medicine, Almazov National Medical Research Centre, Akkuratov st., 2, 197341 St Petersburg, Russia; pliss@niiekf.ru; 7 Department of Normal Physiology, Pavlov First State Medical University of Saint Petersburg, Lev Tolstoy st., 6-8, 197022 St Petersburg, Russia; evlopatina@yandex.ru; 8 Instituto de Medicina y Biología Experimental de Cuyo (IMBECU), Consejo Nacional de Investigaciones Científicas y Técnicas (CONICET), CP 5500 Mendoza, Argentina; diez.emiliano@fcm.uncu.edu.ar; 9 Instituto de Fisiología, Facultad de Ciencias Médicas, Universidad Nacional de Cuyo, Av. Libertador 80, Centro Universitario, CP 5500 Mendoza, Argentina

**Keywords:** melatonin, ischemia, reperfusion, arrhythmia, oxidative stress, depolarization, repolarization

## Abstract

Melatonin is assumed to confer cardioprotective action via antioxidative properties. We evaluated the association between ventricular tachycardia and/or ventricular fibrillation (VT/VF) incidence, oxidative stress, and myocardial electrophysiological parameters in experimental ischemia/reperfusion under melatonin treatment. Melatonin was given to 28 rats (10 mg/kg/day, orally, for 7 days) and 13 animals received placebo. In the anesthetized animals, coronary occlusion was induced for 5 min followed by reperfusion with recording of unipolar electrograms from ventricular epicardium with a 64-lead array. Effects of melatonin on transmembrane potentials were studied in ventricular preparations of 7 rats in normal and “ischemic” conditions. Melatonin treatment was associated with lower VT/VF incidence at reperfusion, shorter baseline activation times (ATs), and activation-repolarization intervals and more complete recovery of repolarization times (RTs) at reperfusion (less baseline-reperfusion difference, ΔRT) (*p* < 0.05). Superoxide dismutase (SOD) activity was higher in the treated animals and associated with ΔRT (*p* = 0.001), whereas VT/VF incidence was associated with baseline ATs (*p* = 0.020). In vitro, melatonin led to a more complete restoration of action potential durations and resting membrane potentials at reoxygenation (*p* < 0.05). Thus, the antioxidative properties of melatonin were associated with its influence on repolarization duration, whereas the melatonin-related antiarrhythmic effect was associated with its oxidative stress-independent action on ventricular activation.

## 1. Introduction

Myocardial ischemia and reperfusion are frequently followed by ventricular tachycardia and/or ventricular fibrillation (VT/VF), which can directly cause sudden cardiac death and as such, presents a major medical and public problem. The development of effective and safe antiarrhythmic drugs is needed for the prevention of fatal tachyarrhythmias in myocardial infarction patients, and pathophysiological targets for these drugs should be identified.

A number of arrhythmogenic mechanisms in ischemic myocardium could be related to an oxidative stress arising during coronary occlusion and especially reperfusion. A so-called “metabolic sink” theory [1,2,3] suggests that superoxide anion flow through the internal membrane anion channels causes collapse of mitochondrial Δψm and a decrease in intracellular adenosine triphosphate (ATP) content, leading to an increase in sarcolemmal ATP-sensitive potassium current (IKATP). The latter results in shortening the action potential duration and locking the membrane potential at the level near potassium reversal potential. Another arrhythmogenic mechanism could be related to inhibition of sodium current (INa) by reactive oxygen species (ROS) [4]. The possible arrhythmogenic consequences of the above mechanisms are conduction-slowing, shortening of the excitation wavelength, and increase of the dispersion of repolarization (DOR), facilitating development of a unilateral conduction block.

Melatonin manifests a multitude of properties throughout the organism. In addition to its proper signaling action via receptor-mediated pathways, melatonin causes the versatile antioxidative effects [5,6] mediated by both ROS-scavenging [7] and stimulation of the antioxidative enzymatic activity [8,9,10]. Melatonin has been proposed to be used in ischemia/reperfusion conditions as a cardioprotective drug [11,12,13,14] and its antiarrhythmic properties were demonstrated [15,16,17,18,19,20,21]. It was shown that melatonin induced a number of electrophysiological effects in myocardium. Specifically, it attenuates the ischemic action potential duration shortening [20] and enhances connexin 43 expression [22]. Both effects may be concerned with the antiarrhythmic action of melatonin.

Mechanisms of cardioprotective, and specifically antiarrhythmic, effects are not clear, but at least part of them are ascribed to its antioxidative properties [14]. However, direct evidence for this melatonin-ROS-arrhythmia chain is lacking. Furthermore, most cardiac electrophysiological effects of melatonin were not studied in situ, i.e., in whole hearts with the realistic hemodynamical load, innervation, and blood supply, factors which may largely affect arrhythmogenesis. It is noteworthy that some of arrhythmogenic prerequisites, such as activation spread and DOR, can be evaluated only in whole hearts or at least relatively large preparations.

In the present study, in the rat myocardial ischemia/reperfusion model, we studied an association between melatonin effects on arrhythmia incidence and spatiotemporal parameters of activation and repolarization. In particular, we tested whether the electrophysiological and/or antiarrhythmic effects of melatonin, if any, are associated with its action on the oxidative stress. Among the descriptors of oxidative state, we studied activity of antioxidants with the hypothesis that melatonin may stimulate it. To this end, we measured a total antioxidant capacity (TAC) and specifically, an activity of superoxide dismutase (SOD), as a cytoplasmic and important mitochondrial antioxidative enzyme. To assess the effects on lipid peroxidation, which can influence the properties of ion channels, a content of 4-hydroxynonenal adducts (HNE) was measured. Finally, a total glutathione (GSH) content was used as a gross estimate of the whole-cell oxidative state, which can reflect the overall effect of melatonin as a ROS-scavenging substance and stimulator of antioxidative systems.

## 2. Results

### 2.1. Spatiotemporal Electrophysiological Parameters in In Vivo Ischemia-Reperfusion Conditions

**Figure 1:**
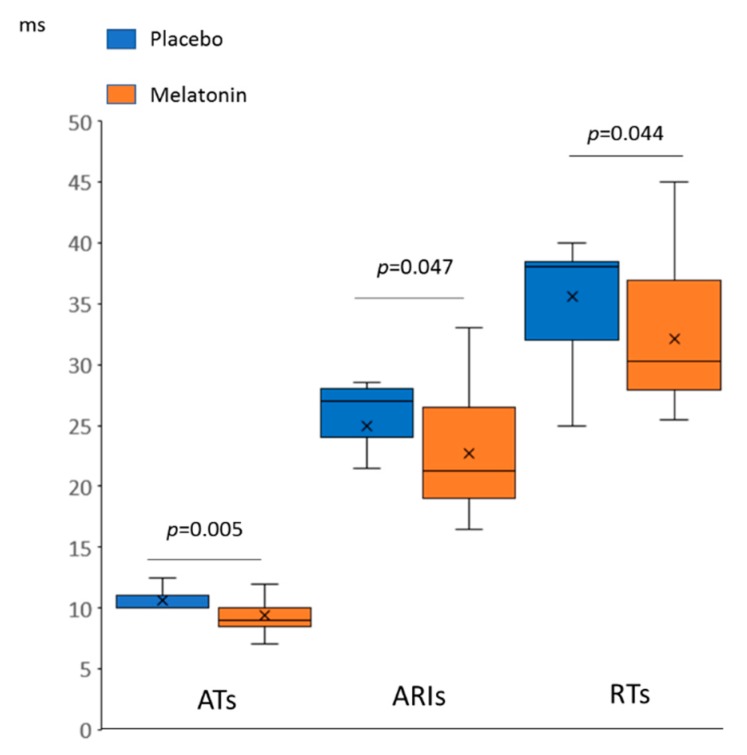
Differences in baseline spatiotemporal electrophysiological parameters between melatonin (*n* = 28) and control (*n* = 13) groups (Mann-Whitney test). AT: activation time, ARI: activation-repolarization interval, RT: repolarization time.

**Figure 2:**
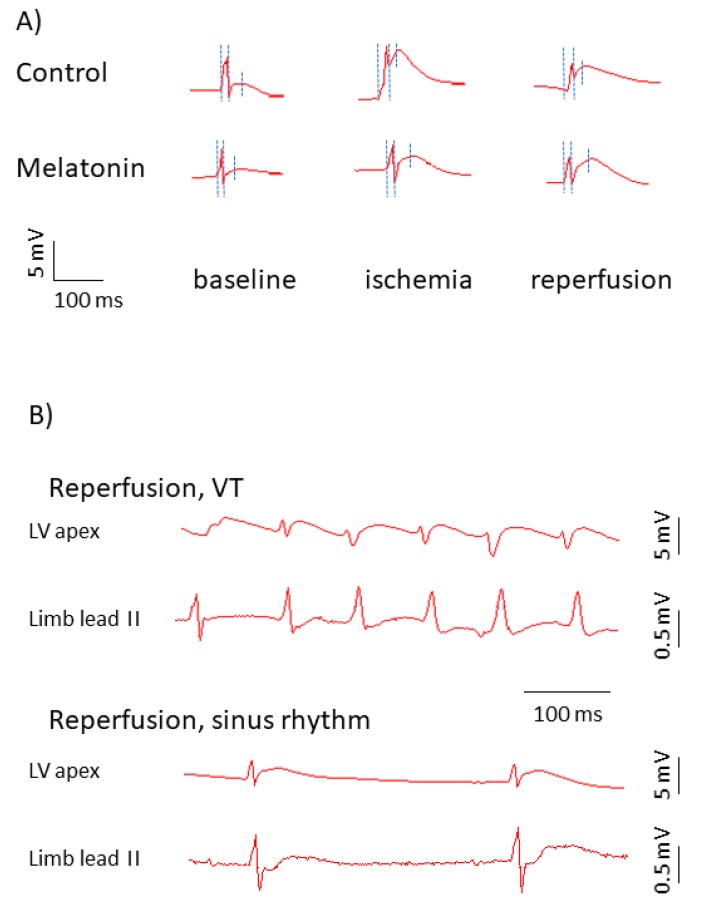
Representative electrograms in control and melatonin-treated animals. (**A**) Representative unipolar electrograms recorded in the affected area (left ventricular (LV) apex). Three dotted vertical lines in each unipolar electrograms identify the QRS onset used as a time-reference point (0 ms), AT and RT from left to right, respectively. ARI is an interval between the second (AT) and third (RT) time-points. Though the ST-segment is extremely short in a typical rat electrocardiogram (ECG), ST-segment elevation and ARI shortening can be seen in ischemia and reperfusion, which indicates electrophysiological changes in the affected area. In the melatonin-treated animal, see a shorter AT in baseline, less pronounced shortening, and more complete restoration of ARI at reperfusion. (**B**) Representative rhythm strips recorded in the affected area and limb lead II in cases of the normal sinus rhythm and in the presence of reperfusion ventricular tachycardia (VT) manifesting as irregular wide QRS complexes and absence of P-waves in the limb lead II.

In the baseline state (Figure 1), the animals treated with melatonin (melatonin group) had shorter activation times (ATs) and activation-repolarization intervals (ARIs), as compared to the untreated animals (control group). Since the end of repolarization time (RT) is the sum AT + ARI (as depicted in Figure 2, panel A), baseline RTs in the melatonin group were also shorter than in the controls. DOR did not differ between the groups (not shown).

**Figure 3:**
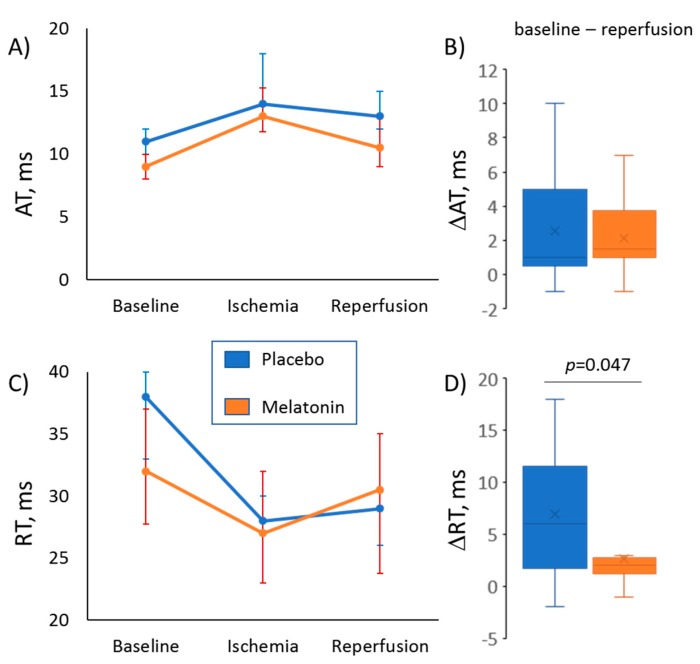
Evolution of activation time (AT, panels **A**, **B**) and repolarization time (RT, panels **C**, **D**) during ischemic episode and subsequent reperfusion in melatonin (*n* = 28) and control (*n* = 13) groups. (Panels **A**, **C**) display changes of median values of ATs and RTs, respectively (error bars are interquartile ranges). (Panels **B**, **D**) show magnitudes of baseline-reperfusion differences in ATs and RTs (∆AT and ∆RT respectively, the reperfusion values subtracted from the baseline values), which express an extent of postischemic recovery of corresponding parameters at reperfusion. Control versus Melatonin comparisons by Mann-Whitney test.

As expected, ischemia resulted in RT shortening and AT prolongation, whereas during reperfusion, these parameters partly restored (Figure 2, panel A and Figure 3). The differences in median ATs between the melatonin and control groups observed in baseline (Figure 1) disappeared during ischemia and regained at reperfusion (10.5 (interquartile range (IQR) 9.0–13.0.

0 ms versus 13 (IQR 11.5–16.0) ms, respectively; *p* = 0.026). As a result, dynamics of the ischemia-reperfusion AT changes were different in the melatonin and control groups (Figure 3, panels A and B). A difference in repolarization dynamics between the groups was more pronounced. The animals given melatonin demonstrated a more complete restoration of RTs during the reperfusion phase, as the difference between baseline and reperfusion values of RTs (ΔRT) was less in the melatonin group (Figure 3, panels C and D).

The median size of ischemia/reperfusion damage area did not differ between melatonin and control groups (96 (IQR 16–100%) versus 84 (IQR 69–99%), *p* = 0.756, respectively). The size of the damage area was associated with the activation delay in the ischemic zone during occlusion in univariate linear regression analysis (regression coefficient 5.47 95% confidence interval (CI) 1.08–9.85; *p* = 0.016). No associations of the size variable were found with neither any of the repolarization parameters nor specifically with VT/VF incidence (odd ratio 1.01 95% CI 0.99–1.03, *p* = 0.235).

### 2.2. Transmembrane Potential Changes in In Vitro Ischemia-Reoxygenation Conditions

**Figure 4:**
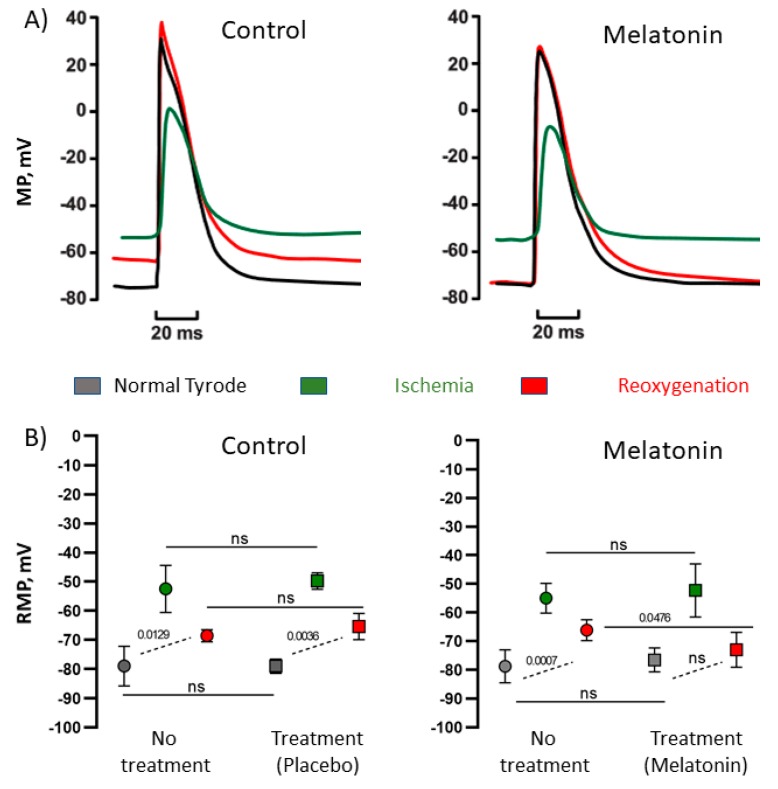
Effects of melatonin on ischemia-induced changes of transmembrane potentials. (**A**) Representative recordings of action potentials in control conditions and in the presence of 10 µM melatonin, see the different extent of recovery at reoxygenation in control and melatonin preparations. (**B**) Resting membrane potential (RMP) in normal, ischemic, and reoxygenation conditions in control preparations and in those with melatonin. Both control and melatonin preparations were subjected to the ischemia/reperfusion cycle twice, first with no treatment and then with placebo or 10 µM melatonin, respectively. See a more complete restoration of RMP at reperfusion in the presence of melatonin. MP: membrane potential, RMP: resting membrane potential.

Ischemia-reoxygenation dynamics of electrophysiological parameters observed in vitro were analogous to those in vivo (Figure 4). In ischemic conditions, transmembrane action and resting potentials changed similarly in the control and melatonin-treated preparations, but at reoxygenation, electrophysiological variables demonstrated more complete restoration or even overcompensation in the presence of 10 µM melatonin (Figure 4, panels A and B). Action potential duration at the level of 90% of repolarization (APD90) shortened in ischemia and prolonged back in reoxygenation. Under melatonin application, the median baseline-reoxygenation difference in APD90 (ΔAPD90) was less than in controls (−8.6 (IQR −9.2–−6.1) ms versus −2.6 (IQR −4.4–−2.2) ms, *p* = 0.031, respectively). The negative sign indicates that APD90 was longer at reoxygenation than at baseline. Resting membrane potential expectedly depolarized in ischemic conditions in both groups; however, in the control group, this parameter remained depolarized at reoxygenation, whereas in the preparations treated with melatonin, resting potential restored nearly completely to the basal value (Figure 4, panel B).

### 2.3. Oxidative Stress Parameters

**Figure 5:**
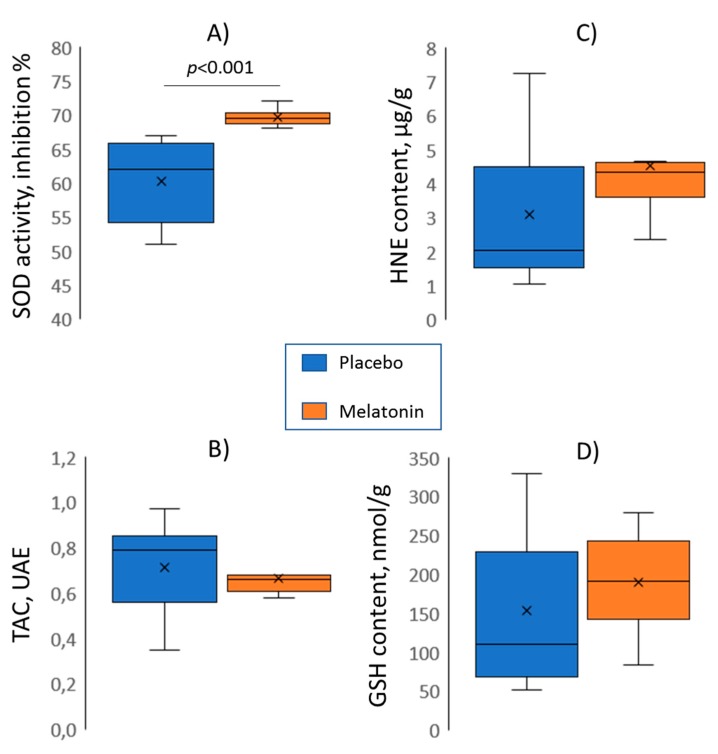
Melatonin (*n* = 10) versus control (*n* = 10) group comparisons (Mann-Whitney tests) of superoxide dismutase activity (SOD, panel **A**), total antioxidant capacity (TAC, panel **B**), 4-hydroxynonenal adducts content (HNE, panel **C**), and total glutathione content (GSH, panel **D**).

Among the oxidative stress parameters (SOD activity, TAC, HNE, GSH) assessed in excised hearts after the ischemic episode and reperfusion, only SOD activity differed between the control and melatonin groups, being higher in the treated animals (Figure 5). We tested the association of the SOD activity with the electrophysiological parameters (Table 1). Among variables differing between the melatonin and control groups, the significant association was found between SOD activity and ΔRT (an index of the resultant ischemia and reperfusion effect on RTs).

### 2.4. Ventricular Tachycardia and/or Ventricular Fibrillation (VT/VF)-Susceptibility

**Figure 6:**
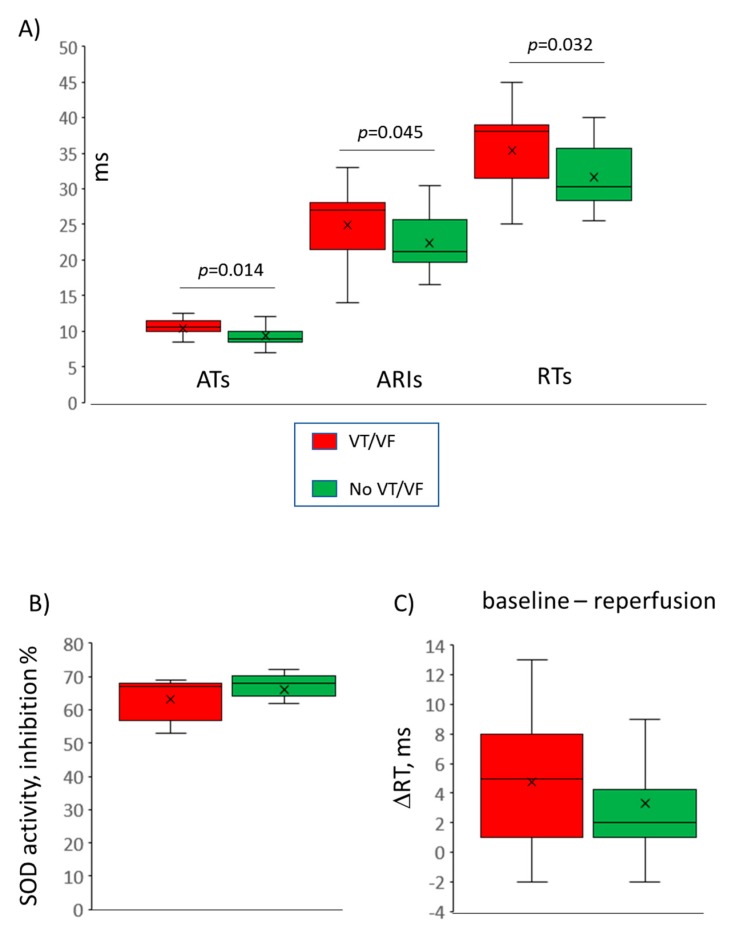
Comparisons between ventricular tachycardia and/or ventricular fibrillation (VT/VF)-susceptible (*n* = 17) versus VT/VF-resistant (*n* = 24) animals. Only variables significantly different between melatonin and control groups are compared here. (**A**) Baseline activation times (ATs), activation-repolarization intervals (ARIs) and repolarization times (RTs), (**B**) superoxide dismutase (SOD) activity, (**C**) baseline-reperfusion differences in RTs (ΔRT) reflecting the extent of postischemic recovery of RT at reperfusion.

A total of 17 rats experienced VT/VF episodes during the first minutes of reperfusion (VT/VF group) and the other 24 animals did not (no VT/VF group). Typical ECGs with and without reperfusion VT are shown in Figure 2, panel B. The melatonin group demonstrated lower VT/VF incidence as compared to the control group (29%, *n* = 28 versus 69%, *n* = 13; *p* = 0.020). In order to establish an arrhythmia-related target for melatonin, the baseline ATs, ARIs, and RTs, ΔRT and SOD activity (i.e., parameters modified by the treatment) were compared in VT/VF versus no VT/VF groups. VT/VF-susceptible rats had longer baseline ATs, ARIs, and RTs as compared to VT/VF-resistant animals, whereas ΔRT and SOD activity did not differ between the groups (Figure 6).

**Figure 7:**
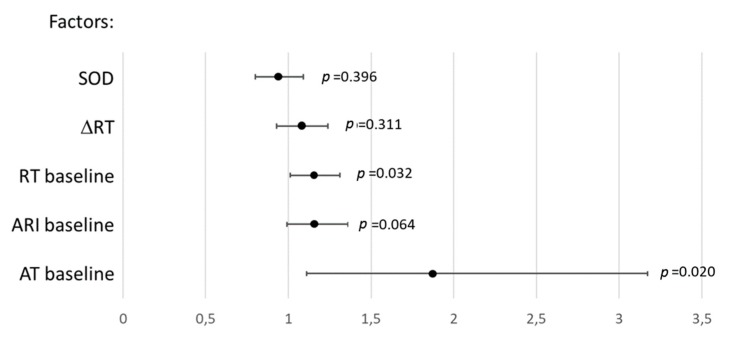
Association (odds ratios and 95% confidence intervals) between ventricular tachycardia and ventricular fibrillation incidence and parameters demonstrating significant differences between the melatonin (*n* = 28) and control (*n* = 13) groups in univariate logistic regression analysis. AT: activation time, RT: repolarization time, ARI: activation-repolarization interval, ΔRT: baseline-reperfusion RT differences, SOD: superoxide dismutase.

In univariate logistic regression analysis, baseline ATs and RTs were associated with VT/VF occurrence (Figure 7). Only baseline ATs were significantly associated with VT/VF incidence in multivariate logistic regression analysis with backward elimination (odd ratio 1.87 95% CI 1.11–3.17, *p* = 0.020) and receiver operating characteristic curve analysis (area under curve 0.727, *p* = 0.014) with the cut-off value of 10.25 ms (sensitivity 0.53, specificity 0.83).

## 3. Discussion

In the present study, we confirmed the previous observations that melatonin confers antiarrhythmic protection in an experimental ischemia/reperfusion model [16,17,18,19,20,21]. As soon as arrhythmias are electrophysiological phenomena, we sought for electrophysiological effects of melatonin relating to its antiarrhythmic action. It was found that chronic (7 days) melatonin administration caused abbreviation of both ATs and ARIs, implying enhancing the activation spread and shortening the action potential duration, respectively. RTs, being the sum of ATs and ARIs, were also decreased in melatonin-treated animals. Furthermore, both the animals chronically given melatonin and the isolated myocardial preparations treated with melatonin, demonstrated more complete (probably faster) restoration of electrophysiological parameters after an ischemic episode.

We evaluated the role of melatonin antioxidative properties in realization of its electrophysiological effects and found an “antioxidative-repolarization relationship”. Consistent with earlier findings [8], we observed the increased SOD activity in the melatonin group of animals, which was probably due to the melatonin-related stimulation of the enzyme. This parameter was associated with the extent of postischemic recovery of RTs, implying a potential causative relation between the oxidative stress and repolarization duration, such as IK(ATP) dependence on ROS accumulation as suggested by the “metabolic sink” theory [1,2,3]. Such a dependence predicts that the higher the antioxidative enzyme activity, the less the ROS accumulation, the less the IK(ATP) current, and the longer the action potential duration after an ischemic episode. However, this chain of events remains to be directly demonstrated. The absence of association between other oxidative stress parameters and melatonin treatment imply either the absence of any melatonin effect on TAC, GSH, and HNE, mutually opposite influences on different components of the oxidative state, or the presence of masking confounding factors. Anyway, only SOD activity demonstrated association with electrophysiological properties of myocardium, which, however, did not relate to arrhythmic susceptibility.

No other associations were found between electrophysiological and oxidative stress variables. Specifically, the differences between treated and untreated animals in activation properties in the baseline state were not related to the studied oxidative stress parameters. The shortened baseline ATs could probably be explained by the known melatonin-related enhancement of connexin 43 expression [22], but this explanation needs to be tested directly. It was exactly the effect on ATs that was associated with the antiarrhythmic action of melatonin. The relationship between arrhythmias and activation implies that the shorter ATs result from the faster conduction, which prevents formation of the reentrant circuit. Melatonin application in vitro enhanced restoration of resting potential after ischemia, which in turn is expected to facilitate conduction via increasing the availability of sodium channels. Consistently with these in vitro observations, the in vivo findings suggest that ATs in melatonin-treated animals restored more completely at reperfusion and the difference in ATs between the treated animals and controls regained. The positive role of shorter ATs was also shown in our recent study of another potential cardioprotective drug with antioxidative properties [23]. It is noteworthy, that in the cited study, baseline AT variations were not even due to the effect of the drug but reflected diverse intrinsic characteristics of myocardial activation in experimental animals.

These findings imply that the antiarrhythmic action of melatonin reported here was independent of antioxidative properties of melatonin which were associated with the changes of repolarization and did not concern activation. In our previous investigation [24], we also observed significant effects of an antioxidative agent on ventricular repolarization. Consistently with our present observations, the effects were related to the magnitude of repolarization duration changes under ischemia/reperfusion, and these effects did not result in the antiarrhythmic action. Though application of antioxidants has been long considered as a promising treatment option on the basis of experimental studies, clinical trials did not support this approach [25].

Limitations of the present study concern the specifics of the rat ischemia/reperfusion model that differs from clinical settings, i.e., the duration of coronary occlusion was only 5 min, and electrophysiological characteristics of the rat myocardium are quite different from the human ones. The number of tests for oxidative stress parameters was inevitably limited, and different relationships between electrophysiological and other oxidative stress variables are not excluded. However, the observation of the SOD activity-repolarization association supports the validity of the present study.

## 4. Materials and Methods

### 4.1. Study Outline

In chronic melatonin- and placebo-treated rats, we induced myocardial ischemia and reperfusion, which was frequently complicated by reperfusion VT/VFs. During ischemia/reperfusion exposure, we recorded ventricular electrograms, from which, data on spatiotemporal activation and repolarization patterns were obtained. Specifically, we evaluated local ATs, RTs, ARIs (the latter served as a surrogate for action potential duration), and DOR. Postmortem, parameters of oxidative stress and antioxidant activity in the hearts were measured. In a separate set of experiments, we tested the effects of melatonin on transmembrane action potential durations in ischemic conditions. Finally, we evaluated the association between VT/VF incidence, oxidative stress, and electrophysiological parameters in melatonin and control groups using linear regression and logistic regression analysis.

### 4.2. Animal In Vivo Experiments

Experiments were done in 41 Wistar male rats (3 months old, body mass 150–200 g). The study conformed to the *Guide for the Care and Use of Laboratory Animals, 8th Edition* published by the National Academies Press (US), 2011, the guidelines from Directive 2010/63/EU of the European Parliament on the protection of animals used for scientific purposes, and was approved by the ethical committee of the Institute of Physiology of the Komi Science Centre, Ural Branch of Russian Academy of Sciences (approval 19 February 2018, amendment 19 September 2018). 28 rats were given melatonin for seven days in single oral daily doses (10 mg/kg/day, at 9.00 am), whereas 13 animals received placebo.

In seven days, acute experiments were performed. For this purpose, the animals were anesthetized with zoletil (ZOLETIL^®^ 100, Virbac S.A., Carros, France, 15 mg/kg, i.m.) and xylazine (XYLA, Interchemie, Castenray, Netherlands, 0,1 mg/kg, i.m.) and mechanically ventilated. The heart was exposed by a midsternal incision and kept warm (37–38 °C) and moistened by irrigation with heated saline. A loose ligature (coated braided polyester, № 5–0, Ti-Cron, Cardiopoint, Dominican Republic) was drawn around a proximal third of the left anterior descending coronary artery (LAD).

Unipolar epicardial electrograms were recorded at baseline, at the end of 5 min LAD occlusion, and at 1 min of reperfusion. Occlusion and reperfusion were done by tightening and loosening of the ligature, respectively. Electrograms were led from a rectangular array of 64 electrodes (8 × 8, 0.5 mm apart) in reference to Wilson’s central terminal from the left ventricular apex (the area supplied by LAD). Standard bipolar limb lead electrocardiograms (ECGs) and the epicardial electrograms were recorded by a custom-designed 144-channel computerized recording system (16 bits; bandwidth 0.05 to 1000 Hz; sampling rate 4000 Hz). At the end of 5 min reperfusion, the heart was immediately excised and frozen in liquid nitrogen. Tissue samples were stored at −45 °C until analysis.

### 4.3. In Vivo Electrophysiological Data Processing

Electrophysiological data from in vivo experiments were processed by the observers blinded to the treatment (placebo or melatonin). Each data record consisted of 64 lead signals, which were inspected for artifacts and low signal/noise ratio. In each lead, an AT (an instant when the activation wave spreading from conduction system reaches the lead site) and RT (an instant when the action potential downstroke reaches its maximal velocity) were determined as time instants of dV/dt min during QRS complex and dV/dt max during T-wave respectively [26], in respect to the onset of QRS complex (Figure 2A). ARI, a surrogate for action potential duration, was calculated as ARI = RT – AT. Values of ATs, RTs, and ARIs were averaged for the given data record. Also, DOR was calculated as the difference between maximal and minimal RTs throughout the same data record. Intensity of a resultant ischemia/reperfusion effect on activation and repolarization was assessed as baseline-reperfusion differences in averaged ATs (ΔAT) and averaged RTs (ΔRT), respectively. The proportion of leads demonstrating ST-segment elevation at onset of reperfusion, calculated as a number of leads with ST-segment elevation divided by the total lead number (64), excluding leads with poor signal quality and expressed in percent, was taken as an estimate for the spatial extent of ischemic damage. As a result, the averaged AT, averaged ARI, averaged RT and DOR for baseline, ischemia and reperfusion time-points, ΔAT, ΔRT, and spatial extent of ischemic damage, were compared between the melatonin and control groups and were tested as electrophysiological predictors of VT/VF.

### 4.4. Oxidative Stress Evaluation

Parameters of oxidative stress were determined by the observer blinded to the treatment (placebo, *n* = 10 or melatonin, *n* = 10). Collagenase type 2, Glutathione Assay kit and Krebs-Henseleit Buffer Modified were purchased from Sigma-Aldrich (St. Louis, MO, USA). The Superoxide Dismutase Activity Assay, HNE Adduct Competitive ELISA Kit, and Total Antioxidant Capacity Assay Kit were purchased from Cell Biolabs (San Diego, CA, USA). The 1× phosphate buffered saline (PBS) (pH 7.4) was used in all experiments. The samples were centrifuged on centrifuge MPW-260R (MPW, Warsaw, Poland). Spectrophotometry was performed using ELISA reader LT-5000MS (Labtech International Ltd., Uckfield, UK). For each analysis, the plates were prepared and calculations were done according to the kit manufacturer’s instructions. For each analysis, the tissue samples of each heart were tested twice, and the average values of two tests were used for statistical evaluation.

#### 4.4.1. Tissue Extracts

For preparation of heart tissue lysate, rat hearts were defrosted at 4 °C above zero and then washed twice in the PBS. Then samples were blotted up and were weighted. Hearts were digested with calcium-free Krebs-Henseleit buffer, which contained 1 mg/mL collagenase type 2 (Sigma-Aldrich, St. Louis, MO 63178, USA). Vials were incubated at 37 °C for 1.5 h. Flaccid samples were washed in the PBS and blotted up. Samples were pressed through the filter membranes. 0.1 g of lysed heart tissue was used for the glutathione assay. The remaining samples of lysed heart tissue (LHT) were diluted by 1 mL of PBS and this solution (LHT solution) was used in other analyses. Samples were weighted after each step of lysed sample preparation and were stored on ice after incubation.

#### 4.4.2. Total Glutathione (GSH) Determination

100 mg of lysed heart tissue were vortexed with 0.3 mL of 5% 5-sulfosalicylic acid. Then, another 0.7 mL of 5% sulfosalicylic acid was added and samples were homogenized until an even suspension was achieved. Samples were incubated at 4 °C for 10 min and then centrifuged at 10,000× *g* for 10 min. Supernatant was measured and immediately used for GSH determination. GSH standard solutions were prepared by serial dilution of the 50 µM GSH solution in 5% sulfosalicylic acid to provide a final concentration of GSH ranging from 3.125 to 50 µM. 10 µL of GSH standards, 5% sulfosalicylic acid (blank), or samples in question were transferred into a 96-well plate. 150 µL of the working mixture solution (assay buffer, enzyme solution, and 5,5′-Dithiobis (2-nitrobenzoic acid) stock solution mixed according to manufacturer’s instruction) was added into each well and mixed by pipetting up and down. The filled plate was incubated at room temperature for 5 min. 50 µL of nicotinamide adenine dinucleotide phosphate solution was added to each well of the plate and mixed by pipetting up and down. Absorbances were measured at 405 nm with kinetic read at 1 min intervals for 5 min. GSH concentration in the samples in question was calculated according to manufacturer’s instructions.

#### 4.4.3. 4-Hydroxynonenal (HNE) Adducts Determination

100 µL of LHT solution was diluted in 1× PBS containing 0.1% bovine serum albumin in a 1:1 ratio and vortexed. All work solutions were prepared according to the kit manufacturer’s instructions. A standard solution in the manufacturer’s assay was diluted in the supplied diluent to a final concentration ranging from 0 to 200 μg/mL. 50 μL of standards (including blank) and samples in question were added into coated wells of a 96-well plate. The plate was incubated at room temperature for 10 min on an orbital shaker. 50 μL of anti-HNE antibody solution was added into each well. The plate was incubated at room temperature for 1 h on an orbital shaker. Then, the wells were washed 3 times with wash buffer. After aspiration, the excess of wash buffer was removed by the tapping of the plate on a paper towel. 100 μL of horseradish peroxidase conjugated secondary antibody solution was added into each well. The plate was incubated at room temperature for 1 h on an orbital shaker. Then, wells were washed 3 times with wash buffer. Excess wash buffer was removed after aspiration by the tapping of the plate on a paper towel. 100 μL of a warm manufacturer’s substrate solution was added into each well. The plate was incubated at room temperature for 15 min on an orbital shaker. 100 μL of a manufacturer’s stop solution was added into each well and the plate was immediately read at 450 nm.

#### 4.4.4. Superoxide Dismutase (SOD) Activity Determination

150 µL of LHT solution was diluted twice by PBS and then centrifuged at 12,000× *g* for 10 min. Tissue lysate supernatant was used in the experiment. Dilution series of SOD standards in the concentration range of 5 U/μL–1.2 mU/μL were prepared according to the kit manufacturer’s instructions. 10 μL of SOD standards (including blank) and SOD samples were added into wells of a 96-well microtiter plate. Then, a mixture of xanthine solution (5 μL), chromagen solution (5 μL), assay buffer (10 μL), and deionized water (60 μL) was added into each well. After that, 10 μL of xanthine oxidase solution was added into each well and mixed. The plate was incubated for 1 h at 37 °C. Absorbances were measured at 490 nm.

#### 4.4.5. Total Antioxidant Capacity (TAC)

LHT solution was centrifuged at 10,000× *g* for 10 min at 4 °C. Tissue lysate supernatant was used in the experiment. 2 mM uric acid standards were prepared immediately before the measurements by the mixing of uric acid powder, 1N NaOH, and deionized water. Dilution series of uric acid standards in the concentration range of 0.0039–1 mM were prepared in deionized water. 20 μL of uric acid standards (including blank) and samples in question were added into each well of the 96-well microtiter plate. Then, 180 μL of a manufacturer’s reaction buffer was added into each well and mixed. Initial absorbance was obtained at 490 nm. 50 μL of a manufacturer’s copper ion reagent was added into each well and incubated for 5 min on an orbital shaker. Then, 50 μL of a manufacturer’s stop solution was added into each well. Absorbances were measured at 490 nm.

### 4.5. In Vitro Transmembrane Potential Recordings

Rats (*n* = 11) were anesthetized with an intraperitoneal injection of 80 mg/kg ketamine and 10 mg/kg xylazine. Heparin (1000 U/kg) was added to the anesthetics solution to prevent blood coagulation in coronary vessels. The chest was opened, and the heart was rapidly excised and rinsed with Tyrode solution of the following composition (mM): NaCl 118.0, KCl 2.7, NaH_2_PO_4_ 2.2, MgCl_2_ 1.2, CaCl_2_ 1.2, NaHCO_3_ 25.0, glucose 11.0, bubbled with carbogen (95% O_2_ + 5% CO_2_), pH 7.4 ± 0.1 (≈303 mOsm/L). Right ventricular wall preparation was isolated and pinned with endocardial side up to the bottom of a 3 mL chamber continuously perfused with Tyrode solution (10 mL/min, 37.5 °C). The constant electrical pacing with a pair of silver electrodes (2 ms rectangular impulses with amplitude twice above a threshold and 4 Hz pacing rate) was started immediately after the preparation. Before the experiment, the preparations were equilibrated in the perfusion chamber for 30 min.

Transmembrane potentials (resting potential and electrically-evoked action potentials) in the right ventricular preparations were recorded using the standard microelectrode technique. Glass microelectrodes (resistance 10–15 MOm) connected to an amplifier (a-M system 1600, Washington DC, USA) were used for registration. The amplified signal was digitized by Analog-to-digital converter (ADC, E-154, L-card, Moscow, Russia) and then recorded and processed using the Power Graph 3.3.8 software (Disoft, Moscow, Russia). The electrical activity was registered from the endocardial side of the ventricular wall continuously during the experiment.

In the first part of experiment (no treatment), transmembrane potentials were recorded in the preparations perfused with Tyrode solution of normal composition during 5 min (“normal” state). Then, the preparations were exposed to «ischemic» conditions, which ensued from changing the Tyrode solution composition (mM: NaCl 118.0, KCl 10.0, NaH_2_PO_4_ 2.2, MgCl_2_ 1.2, CaCl_2_ 1.2, NaHCO_3_ 9.0, glucose 11., ≈ 285 mOsm/L, without oxygenation) for 5 min (“ischemia” state). After that, the preparations were reperfused with normal Tyrode solution (“reoxygenation” state) for 5 min. The second part of experiment (treatment with melatonin or placebo) was performed after 30 min perfusion of the preparations with normal Tyrode solution. The electrical activity was registered as in the first part of experiment, but with the addition of placebo (*n* = 4) or 10 µM melatonin (Sigma-Aldrich, St. Louis, MO 63178, USA) (*n* = 7) to each solution. Before the registration of the “normal” action potential, the treatment solution had been applied for 10 min. Placebo “treatment” was done in order to take into account the possible effects of preconditioning by preceding ischemia and time-dependent rundown of the preparations. Action potential duration at the level of 90% of repolarization (APD90) and resting membrane potential were calculated and analyzed at the fifth minute of each experimental step.

### 4.6. Statistical Analysis

Data are expressed as medians and interquartile intervals. Statistical analysis was performed with the SPSS package (IBM SPSS Statistics 23). The Mann-Whitney test was used to compare groups of animals. Wilcoxon and Friedman tests were applied for paired and multiple comparisons within the same groups, respectively. VT/VF incidence in melatonin-treated and untreated animals was compared using the Chi-square test. The association of myocardial depolarization and repolarization parameters with VT/VF occurrence and parameters of oxidative stress was assessed by univariate and multivariate linear and logistic regression analyses and ROC curve analysis. The differences were considered significant at *p* < 0.05.

## 5. Conclusions

In the present investigation we demonstrated electrophysiological, antioxidative, as well as antiarrhythmic effects of melatonin in an experimental ischemia/reperfusion model. It was found that the antiarrhythmic action was associated with the antioxidative-independent melatonin influence on ventricular activation, whereas the enhancement of SOD activity correlated with the melatonin effects on ventricular repolarization.

## Figures and Tables

**Table 1:** Associations between superoxide dismutase (SOD) activity and spatiotemporal electrophysiological parameters in univariate linear regression analysis.

Variable	Regression Coefficient	95% CI	*p* Value
AT baseline *	0.02	−0.06–0.09	0.663
ARI baseline *	0.02	−0.17–0.21	0.832
RT baseline *	0.04	−0.18–0.25	0.731
AT reperfusion *	−0.11	−0.28–0.06	0.172
ARI reperfusion	0.93	0.44–1.41	0.001
RT reperfusion	0.90	0.30–1.50	0.006
ΔAT (baseline–reperfusion)	−0.44	−1.62–0.74	0.441
ΔRT (baseline–reperfusion) *	−0.55	−0.85–−0.25	0.001

* *p* < 0.05 between melatonin-treated (*n* = 10) and untreated (*n* = 10) animals; AT: activation time, RT: repolarization time, ARI: activation-repolarization interval, ΔAT: baseline-reperfusion AT differences, ΔRT: baseline-reperfusion RT differences, CI- confidence interval.

## Abbreviations

VT/VF	ventricular tachycardia/fibrillation
AT	activation time
RT	repolarization time
ARI	activation-repolarization interval
DOR	dispersion of repolarization
LAD	left anterior descending coronary artery
IQR	interquartile range
ECG	electrocardiogram
CI	confidence interval
GSH	total glutathione
TAC	total antioxidant capacity
HNE	4-hydroxynonenal
SOD	superoxide dismutase
LHT	lysed heart tissue
PBS	phosphate buffered saline
APD	action potential duration
